# Severe Hypophosphatemia and Renal Phosphate Wasting Presenting as Atraumatic Bilateral Hip Pain: Suspected Hypophosphatemic Osteomalacia

**DOI:** 10.7759/cureus.111336

**Published:** 2026-06-23

**Authors:** Adonai James Chery, Atta Ur Rehman Ilham

**Affiliations:** 1 Medicine and Surgery, Escuela Latinoamericana de Medicina, Port-au-Prince, HTI; 2 Orthopaedics and Trauma, Northwick Park Hospital, London, GBR

**Keywords:** atraumatic hip pain, bilateral hip pain, fgf23, hypophosphatemia, metabolic bone disease, orthopedic diagnosis, osteomalacia, phosphate wasting disorder, phosphate-wasting osteomalacia, renal phosphate wasting

## Abstract

Hypophosphatemic osteomalacia is an uncommon metabolic bone disorder that may present with nonspecific musculoskeletal symptoms, resulting in delayed diagnosis across orthopedic and emergency care pathways. We describe a 55-year-old man who presented through a National Health Service (NHS) orthopedic pathway with progressive atraumatic bilateral hip and groin pain, worsening mobility, proximal muscle weakness, and functional decline, initially managed as presumed mechanical musculoskeletal pain. Plain hip and pelvic radiographs did not identify a structural explanation for the severity of his symptoms.

Initial laboratory investigations demonstrated profound hypophosphatemia (0.32 mmol/L) with markedly elevated alkaline phosphatase (428 IU/L), mildly reduced corrected calcium (2.18 mmol/L), elevated parathyroid hormone, and vitamin D insufficiency. Because the severity of hypophosphatemia appeared disproportionate to the degree of vitamin D deficiency, extended metabolic bone investigations were undertaken. Further testing demonstrated renal phosphate wasting with reduced tubular maximum phosphate reabsorption corrected for glomerular filtration rate (TmP/GFR 0.42 mmol/L) and C-terminal fibroblast growth factor 23 (FGF23) above the laboratory reference range (248 RU/mL), supporting suspected FGF23-mediated phosphate-wasting osteomalacia; localization imaging and definitive etiologic classification remained pending at the time of reporting.

Severe hypophosphatemia with elevated alkaline phosphatase in patients presenting with unexplained atraumatic bilateral hip or groin pain, proximal weakness, or progressive mobility impairment should prompt consideration of metabolic bone disease and renal phosphate wasting. This case also illustrates that clinically significant osteomalacia may occur despite only mildly reduced calcium concentrations and initially nondiagnostic radiographs. Early biochemical recognition may facilitate timely specialist referral and help reduce diagnostic delay and downstream skeletal morbidity in patients with phosphate-wasting disorders.

## Introduction

Osteomalacia is a metabolic bone disorder characterized by impaired mineralization of mature osteoid tissue, resulting in skeletal fragility, musculoskeletal pain, proximal muscle weakness, impaired mobility, and insufficiency fractures [1]. Although vitamin D deficiency remains the most common global cause, chronic phosphate depletion and renal phosphate-wasting disorders are increasingly recognized causes of adult hypophosphatemic osteomalacia in endocrine and metabolic bone practice [2,3].

Phosphate plays a central role in hydroxyapatite formation and normal skeletal mineralization. Persistent hypophosphatemia impairs mineral deposition and promotes accumulation of unmineralized osteoid, leading to bone pain, gait dysfunction, proximal myopathy, and increased fracture susceptibility [3]. Chronic phosphate depletion may occur secondary to inherited disorders such as X-linked hypophosphatemia (XLH), acquired fibroblast growth factor 23 (FGF23)-mediated conditions such as tumor-induced osteomalacia (TIO), renal tubular dysfunction, medication exposure, including intravenous iron therapy, or gastrointestinal malabsorption disorders [2,3].

The clinical presentation is frequently nonspecific and may mimic common orthopedic or rheumatologic conditions, leading patients to be initially assessed for chronic hip osteoarthritis, lumbar radiculopathy, degenerative musculoskeletal disease, inflammatory arthropathy, or fibromyalgia [4]. Consequently, many patients experience prolonged diagnostic delays and undergo repeated orthopedic, rheumatologic, neurologic, physiotherapy, or emergency assessments before metabolic bone disease is recognized [3,4].

Biochemical assessment is therefore central to diagnosis. Current approaches to suspected phosphate-wasting osteomalacia emphasize evaluation of serum phosphate, calcium, alkaline phosphatase (ALP), parathyroid hormone, vitamin D status, renal function, and formal assessment of renal phosphate handling, including tubular maximum phosphate reabsorption corrected for glomerular filtration rate (TmP/GFR) [2,3]. FGF23 measurement can assist diagnostic evaluation, but interpretation is assay-specific because intact and C-terminal assays are not interchangeable, and results should be interpreted using the laboratory-specific reference range [5]. In practice, an elevated or inappropriately nonsuppressed FGF23 result is most meaningful when interpreted alongside persistent hypophosphatemia, elevated ALP, calcium and PTH status, vitamin D status, renal function, and evidence of renal phosphate wasting such as reduced TmP/GFR [2,3,5]. Importantly, clinically significant osteomalacia may occur despite only mildly reduced or near-normal calcium concentrations; therefore, normal or near-normal calcium should not be falsely reassuring when phosphate is low, ALP is elevated, and symptoms include atraumatic bone pain, proximal weakness, or gait dysfunction [1,3].

Within orthopedic and emergency care pathways, serum phosphate may be overlooked during routine musculoskeletal assessment despite phosphate depletion being a potentially treatable cause of skeletal morbidity [3,4]. Elevated ALP in the setting of unexplained atraumatic musculoskeletal pain, proximal weakness, gait dysfunction, or impaired mobility should prompt consideration of metabolic bone disease and renal phosphate wasting, particularly when symptoms appear disproportionate to routine structural findings.

The relevance of adult phosphate-wasting disorders in the United Kingdom (UK) has increased following National Institute for Health and Care Excellence (NICE) guidance recommending burosumab as an option for adults with XLH [6-8]. However, diagnostic evaluation of suspected FGF23-mediated osteomalacia remains complex and requires careful differentiation between hereditary and acquired causes of phosphate wasting.

We present a case of severe hypophosphatemia with renal phosphate wasting identified during orthopedic assessment of progressive atraumatic bilateral hip and groin pain, highlighting the diagnostic importance of biochemical pattern recognition in unexplained musculoskeletal presentations within acute orthopedic pathways.

## Case presentation

A 55-year-old man presented to the emergency department with progressive atraumatic bilateral hip and groin pain associated with worsening mobility over several months. Symptoms were exacerbated by weight-bearing activity and stair climbing and were associated with generalized fatigue, proximal lower limb weakness, difficulty rising from a seated position, and progressively reduced walking tolerance. There was no preceding trauma or clear inciting mechanical injury.

Past medical history was otherwise unremarkable, with no known chronic kidney disease, inflammatory arthropathy, malignancy, prior metabolic bone disease, or previous fragility fractures. There was no history of corticosteroid exposure, anticonvulsant therapy, alcohol excess, bariatric surgery, chronic gastrointestinal malabsorption, recent intravenous iron therapy, known nutritional deficiency, restrictive diet, or prolonged immobility. The patient denied childhood lower limb deformity, recurrent dental abscesses, or a known family history of hereditary phosphate-wasting disorders.

Examination demonstrated an antalgic gait with proximal lower limb weakness and reduced functional mobility. There was no focal joint erythema, inflammatory synovitis, distal neurologic deficit, or red-flag spinal findings. Hip movements were uncomfortable bilaterally during weight-bearing and functional activity assessment, although no gross deformity was identified.

**Figure 1:**
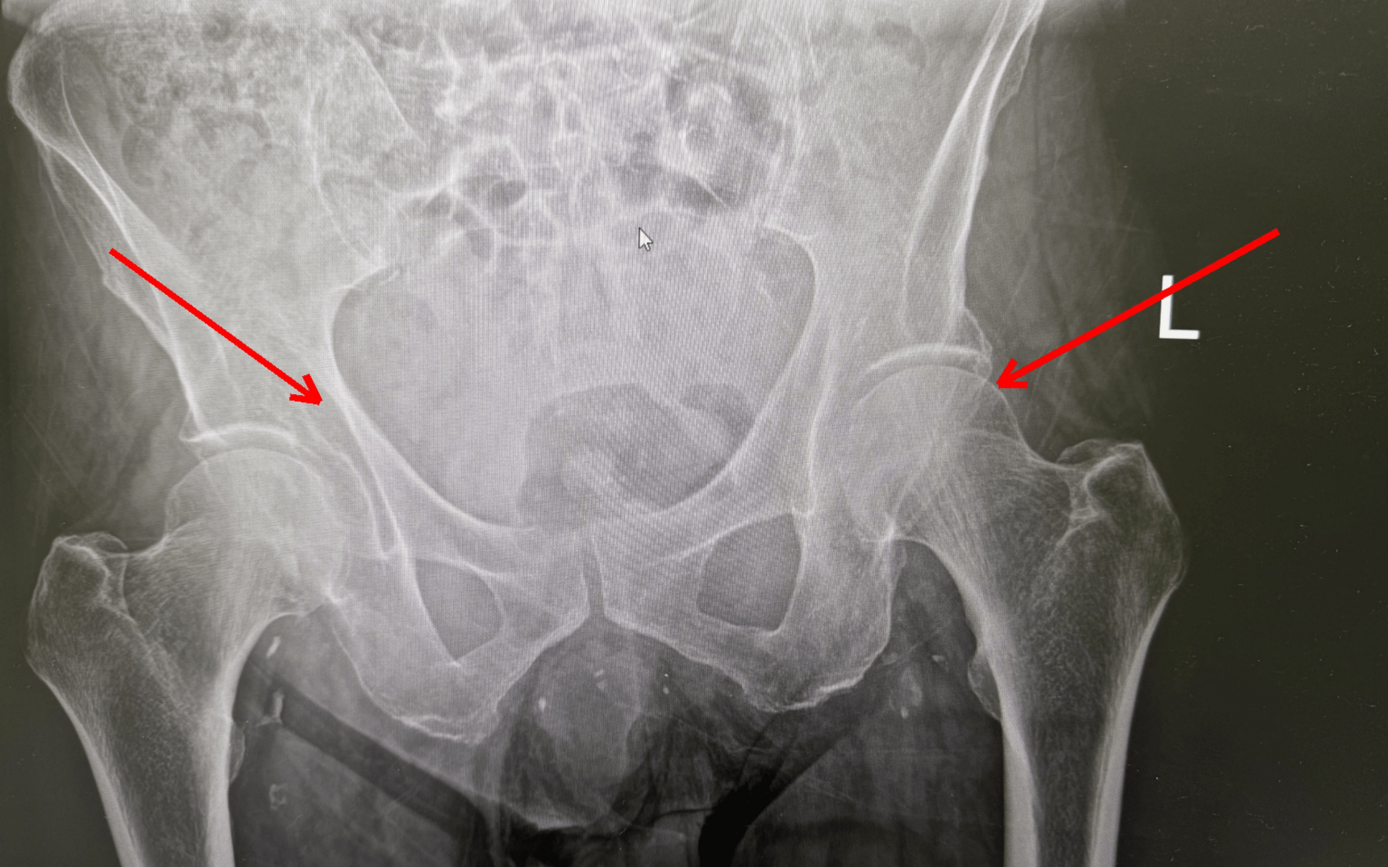
Initial plain pelvic and bilateral hip radiograph Initial plain pelvic and bilateral hip radiograph obtained during orthopedic assessment. Straight digital arrows indicate the clinically symptomatic bilateral hip regions. No acute fracture, dislocation, or clear structural abnormality was identified to account for the severity of symptoms.

Initial orthopedic assessment favored a mechanical musculoskeletal etiology because of the preserved distal neurologic examination and the absence of overt inflammatory or traumatic features. Plain radiographic imaging of the pelvis and hips did not demonstrate acute fracture, dislocation, or other clear structural pathology to account for the severity of the symptoms. The initial plain pelvic and bilateral hip radiographs are shown in Figure 1. However, routine biochemical investigations demonstrated profound hypophosphatemia with markedly elevated alkaline phosphatase (ALP), prompting consideration of metabolic bone disease. Initial laboratory investigations are summarized in Table 1. 

**Table 1:** Initial biochemical investigations at presentation Values are from routine laboratory testing at presentation. ALP: alkaline phosphatase, PTH: parathyroid hormone, eGFR: estimated glomerular filtration rate, CRP: C-reactive protein.

Investigation	Result	Reference range
Serum phosphate	0.32 mmol/L	0.80-1.50 mmol/L
ALP	428 IU/L	30-130 IU/L
Corrected calcium	2.18 mmol/L	2.20-2.60 mmol/L
Parathyroid hormone (PTH)	9.2 pmol/L	1.6-6.9 pmol/L
25-hydroxyvitamin D	42 nmol/L	>50 nmol/L
Magnesium	0.78 mmol/L	0.70-1.00 mmol/L
Creatinine	79 µmol/L	64-104 µmol/L
Estimated glomerular filtration rate (eGFR)	>90 mL/min/1.73 m²	>60 mL/min/1.73 m²
C-reactive protein (CRP)	8 mg/L	<5 mg/L

Standardized serial fasting phosphate and ALP measurements before treatment initiation were not available in the case record. Historical pre-symptom phosphate and ALP values were also not available. Therefore, the initial severe hypophosphatemia and elevated ALP were interpreted cautiously in combination with the broader biochemical pattern, including calcium, PTH, vitamin D status, preserved renal function, paired serum and urine phosphate/creatinine measurements, and subsequent TmP/GFR assessment. Although serum phosphate may be influenced by fasting status and diurnal variation, the phosphate concentration of 0.32 mmol/L was markedly below the local adult reference range of 0.80-1.50 mmol/L and less than half the lower limit of normal. This degree of hypophosphatemia appeared disproportionate to the relatively modest reduction in 25-hydroxyvitamin D concentration, which was 42 nmol/L against a laboratory sufficiency threshold of >50 nmol/L. Although vitamin D insufficiency and secondary hyperparathyroidism may have contributed to the biochemical abnormalities, the severity of hypophosphatemia and the reduced TmP/GFR raised concern for a phosphate-wasting disorder rather than isolated nutritional vitamin D insufficiency alone. Extended metabolic bone investigations were therefore undertaken.

Further investigations demonstrated renal phosphate wasting despite severe hypophosphatemia. Tubular maximum phosphate reabsorption corrected for glomerular filtration rate (TmP/GFR) was reduced at 0.42 mmol/L, supporting impaired renal phosphate handling. C-terminal FGF23 was above the laboratory reference range at 248 RU/mL, which supported, but did not by itself confirm, an FGF23-mediated phosphate-wasting process. Definitive etiologic classification and localization investigations remained pending at the time of reporting. Extended phosphate metabolism investigations are summarized in Table 2. TmP/GFR was calculated from paired serum and urine phosphate and creatinine measurements obtained during the same sampling period. Fractional tubular reabsorption of phosphate was first derived using the standard relationship:

$\text{Fractional tubular reabsorption of phosphate} = 1 &minus; \frac{\text{Urine phosphate}\times \text{Serum creatinine}}{\text{Serum phosphate}\times \text{Urine creatinine}}$

TmP/GFR was then derived from serum phosphate and fractional tubular phosphate reabsorption using standard equation-based or nomogram methods. 

**Table 2:** Extended investigations supporting renal phosphate wasting Extended investigations demonstrated reduced TmP/GFR with supportive biochemical findings for renal phosphate wasting. TmP/GFR: tubular maximum phosphate reabsorption corrected for glomerular filtration rate.

Investigation	Result	Reference range	Interpretation
TmP/GFR	0.42 mmol/L	0.80-1.35 mmol/L	Impaired renal phosphate reabsorption
FGF23 (C-terminal assay)	248 RU/mL	<180 RU/mL	Above assay-specific reference range; supports but does not confirm FGF23-mediated phosphate wasting
Serum bicarbonate	24 mmol/L	22-29 mmol/L	Renal tubular acidosis not supported
Urinary glucose	Negative	Negative	Glycosuria absent; Fanconi syndrome not supported
Serum electrophoresis	Normal	—	No paraprotein detected

The overall clinical and biochemical picture was most consistent with suspected phosphate-wasting osteomalacia, with FGF23-mediated disease high on the differential diagnosis. In a 55-year-old man without documented childhood skeletal deformity, recurrent dental abscesses, or a family history of phosphate-wasting disorders, an acquired cause such as tumor-induced osteomalacia is a more plausible working diagnosis than XLH, although mild or previously unrecognized hereditary disease cannot be excluded without specialist evaluation. Alternative causes, including Fanconi syndrome, renal tubular acidosis, plasma cell dyscrasia, and isolated nutritional osteomalacia, were considered less likely based on the available biochemical profile. However, definitive differentiation between acquired and hereditary phosphate-wasting disorders remained pending at the time of reporting.

The case-specific clinical and biochemical features that redirected the diagnostic pathway from an initially presumed mechanical musculoskeletal etiology toward suspected phosphate-wasting osteomalacia are summarized in Table 3. 

**Table 3:** Case-specific diagnostic reasoning supporting suspected phosphate-wasting osteomalacia This table summarizes the clinical and biochemical features that redirected evaluation from an initially presumed mechanical musculoskeletal etiology toward suspected phosphate-wasting osteomalacia. Interpretations are based on the available investigations at the time of reporting, while definitive etiological classification remained pending. ALP: alkaline phosphatase; FGF23: fibroblast growth factor 23; RTA: renal tubular acidosis; TmP/GFR: tubular maximum phosphate reabsorption corrected for glomerular filtration rate; XLH: X-linked hypophosphatemia.

Clinical or biochemical feature	Finding in this case	Diagnostic significance
Pattern of pain	Progressive atraumatic bilateral hip and groin pain worsened by weight-bearing and stair climbing	Bilateral atraumatic symptoms with functional decline were disproportionate to an isolated focal mechanical explanation
Functional features	Worsening mobility, proximal lower limb weakness, difficulty rising from a seated position, and reduced walking tolerance	Proximal weakness and gait dysfunction supported possible metabolic bone disease rather than isolated local joint pathology
Initial radiographs	Plain pelvic and hip radiographs did not show acute fracture, dislocation, or a clear structural explanation	Non-diagnostic radiographs did not exclude early osteomalacia or insufficiency injury; biochemical abnormalities became the key diagnostic clue
Serum phosphate	0.32 mmol/L	Profound hypophosphatemia supported a phosphate-depletion state capable of impairing bone mineralization
Alkaline phosphatase	428 IU/L	Markedly elevated ALP supported increased bone turnover or defective mineralization and argued against an isolated mechanical explanation
Corrected calcium	2.18 mmol/L	Only mildly reduced calcium showed that clinically significant osteomalacia can occur without marked hypocalcemia
25-hydroxyvitamin D	42 nmol/L	Mild vitamin D insufficiency may have contributed but was unlikely to fully explain the depth of hypophosphatemia
Parathyroid hormone	9.2 pmol/L	Secondary hyperparathyroidism may have contributed to phosphate loss but did not fully explain the renal phosphate-wasting pattern
TmP/GFR	0.42 mmol/L	Reduced renal phosphate reabsorption supported inappropriate renal phosphate wasting despite severe hypophosphatemia
C-terminal FGF23	248 RU/mL	Above the assay-specific reference range, supporting suspected FGF23-mediated phosphate wasting while not being definitive alone
Fanconi syndrome / renal tubular acidosis screen	Normal bicarbonate and negative urinary glucose	These findings made renal tubular acidosis and Fanconi syndrome less supported
Plasma cell screen	Normal serum electrophoresis	Plasma cell dyscrasia was less likely based on available testing
Etiological classification	Adult onset, no childhood deformity, no recurrent dental abscesses, and no known family history	Acquired phosphate-wasting disease, such as tumor-induced osteomalacia was a plausible working diagnosis, although definitive classification remained pending

The patient was commenced on oral phosphate supplementation together with active vitamin D therapy under endocrine and metabolic bone specialist supervision. The exact initial dose titration schedule and serial post-treatment biochemical values were not available in the case record. Treatment was therefore described as specialist-guided, with planned monitoring of serum phosphate, calcium, ALP, PTH, renal function, and urinary calcium excretion to balance symptomatic and biochemical improvement against the risks of hypercalcemia, hypercalciuria, nephrocalcinosis, and gastrointestinal intolerance. Early clinical follow-up suggested improvement in pain and mobility after treatment initiation, although objective biochemical reassessment and definitive etiologic evaluation were still ongoing at the time of reporting. Further characterization of the underlying phosphate-wasting disorder, including consideration of localization studies and possible genetic investigations, was planned through specialist metabolic bone follow-up.

## Discussion

This case highlights the diagnostic importance of biochemical pattern recognition in patients presenting with unexplained atraumatic orthopedic pain, particularly the combination of severe or persistent hypophosphatemia, elevated ALP, and normal or only mildly reduced calcium in the presence of proximal weakness, gait dysfunction, or symptoms disproportionate to routine imaging findings. Although structural imaging frequently dominates musculoskeletal assessment pathways, metabolic bone disease may initially become apparent through biochemical abnormalities before a definitive structural explanation for symptoms has been established [1,4]. In the present case, recognition of profound hypophosphatemia, markedly elevated alkaline phosphatase (ALP), proximal muscle weakness, and renal phosphate wasting prompted metabolic bone evaluation during an orthopedic assessment pathway that had initially favored a mechanical musculoskeletal etiology.

A key learning point from this case is that clinically significant osteomalacia may occur despite only mildly reduced or near-normal corrected calcium concentrations. In phosphate-wasting disorders, chronic phosphate depletion rather than calcium deficiency represents the principal mechanism impairing skeletal mineralization [2]. Consequently, clinicians focusing primarily on calcium or vitamin D concentrations may overlook clinically important metabolic bone disease despite progressive musculoskeletal symptoms and functional decline [1,3].

The markedly elevated ALP observed in this patient was interpreted as a supportive but nonspecific biochemical clue. ALP elevation may arise from bone, hepatic, or other sources; bone-specific ALP, ALP isoenzyme fractionation, and contemporaneous liver biochemistry sufficient to confirm a nonhepatic ALP source were not available in the case record. Therefore, the ALP result was not interpreted in isolation but alongside profound hypophosphatemia, proximal weakness, atraumatic weight-bearing pain, nondiagnostic plain radiographs, and reduced TmP/GFR. In this clinical and biochemical context, the elevated ALP supported increased bone turnover or defective skeletal mineralization and argued against an isolated mechanical orthopedic explanation [1,3,4]. Elevated ALP in the setting of unexplained atraumatic musculoskeletal pain should therefore prompt consideration of phosphate metabolism and metabolic bone disease, particularly when symptoms appear disproportionate to examination findings or are associated with proximal weakness, gait dysfunction, or impaired mobility [1,3,4].

Vitamin D insufficiency and secondary hyperparathyroidism may have contributed to phosphate loss in this case; however, the severity of hypophosphatemia, markedly reduced TmP/GFR, and C-terminal FGF23 above the assay reference range suggested an additional phosphate-wasting process. Because the assay used was C-terminal and iron studies were unavailable, the FGF23 result should be interpreted as supportive rather than definitive evidence of FGF23-mediated disease [5,9]. In this context, reduced TmP/GFR was particularly important because assessment of renal phosphate handling is central to distinguishing renal phosphate wasting from nonrenal causes of hypophosphatemia [10].

FGF23-mediated disorders are well-recognized causes of adult hypophosphatemic osteomalacia. FGF23 suppresses renal phosphate reabsorption within the proximal renal tubule and reduces calcitriol synthesis, ultimately producing chronic phosphate depletion and impaired skeletal mineralization [2]. In the present case, the late age at presentation and absence of documented childhood skeletal deformity, recurrent dental abscesses, or a family history make an acquired disorder such as tumor-induced osteomalacia a plausible working diagnosis, although hereditary disease cannot be excluded without specialist evaluation. Recent literature on tumor-induced osteomalacia emphasizes that delayed recognition is common because symptoms may resemble nonspecific musculoskeletal or rheumatologic disease and that localization and definitive etiologic classification may require specialist evaluation [11,12].

This case was diagnostically challenging because the presenting features resembled common mechanical orthopedic pathology. In patients with bilateral atraumatic hip or groin pain, proximal weakness, elevated ALP, and symptoms disproportionate to routine imaging findings, metabolic bone disease and phosphate wasting should be considered early to reduce repeated assessments and diagnostic delay.

Recent NICE guidance has recommended burosumab as an option for adults with XLH in the UK [7,8]. That development is relevant to specialist metabolic bone practice, but it should not be used to imply a treatment indication in an unclassified phosphate-wasting disorder such as the present case.

This report has several limitations. The case is best interpreted as suspected rather than definitively confirmed FGF23-mediated osteomalacia because localization imaging and definitive etiologic classification remained pending at the time of reporting. Although the biochemical profile supported renal phosphate wasting, further evaluation, including possible genetic or localization investigations, was ongoing. In suspected tumor-induced osteomalacia, specialist-directed localization may include functional imaging such as somatostatin receptor PET/CT, including DOTATATE PET/CT where available, followed by targeted anatomic imaging with MRI or CT to define a candidate lesion and guide management [11,12]. Additional metabolic bone investigations, including 1,25-dihydroxyvitamin D measurement and comprehensive iron studies, were not available at the time of reporting. Nevertheless, the available biochemical abnormalities were sufficient to redirect the diagnostic pathway, prompt specialist referral, and justify interim specialist-guided management while etiologic workup continued. Only initial plain pelvic and hip radiographs were available at the time of reporting; these do not exclude early osteomalacia-related pseudofractures or insufficiency injuries. Additional skeletal and etiologic localization imaging remained pending.

## Conclusions

Hypophosphatemic osteomalacia should be considered in patients presenting with unexplained atraumatic bilateral hip or groin pain, proximal muscle weakness, gait dysfunction, or musculoskeletal symptoms disproportionate to presumed mechanical pathology. This case demonstrates that severe hypophosphatemia with elevated alkaline phosphatase may provide an important biochemical clue to underlying metabolic bone disease, even when initial radiographs do not identify a clear structural explanation. Within orthopedic and emergency care pathways, early serum phosphate measurement may help identify renal phosphate-wasting disorders sooner, particularly when pain is bilateral, associated with proximal weakness or impaired mobility, or accompanied by elevated alkaline phosphatase. Although definitive etiologic classification remained pending at the time of reporting, early biochemical recognition of renal phosphate wasting redirected the diagnostic pathway, facilitated specialist metabolic bone evaluation, and supported initiation of specialist-guided treatment.
